# An assessment of healthcare professionals’ knowledge about and attitude towards influenza vaccination in Freetown Sierra Leone: a cross-sectional study

**DOI:** 10.1186/s12889-017-4700-2

**Published:** 2017-09-05

**Authors:** Peter Bai James, Inayat Ur Rehman, Abudulai Jawo Bah, Michael Lahai, Christine Princess Cole, Tahir Mehmood Khan

**Affiliations:** 1 Faculty of Pharmaceutical Sciences, College of Medicine and Allied Health Sciences University of Sierra Leone, Connaught Teaching Hospital, Freetown, Sierra Leone; 2 School of Pharmacy, Monash University Malaysia, 45700 Bandar Sunway, Selangor Malaysia; 3 Department of Pharmacy, Abdul Wali Khan University Mardan, Mardan, Pakistan; 4 Faculty of Basic Medical sciences, College of Medicine and Allied Health Sciences University of Sierra Leone, Connaught Teaching Hospital, Freetown, Sierra Leone

**Keywords:** Healthcare professionals, Influenza, Vaccination, Knowledge, Attitude, Sierra Leone

## Abstract

**Background:**

Vaccinating healthcare professionals against influenza is considered an effective infection control measure. However, there is a low uptake of influenza vaccine among healthcare professionals around the globe. Currently, it is unknown whether healthcare professionals in Sierra Leone are aware of, and have been vaccinated against influenza. Also, there is a paucity of research evidence on their level of knowledge and attitude toward influenza vaccination. This study assessed healthcare professionals’ current influenza vaccine uptake rate, reasons for not getting vaccinated as well as their awareness, knowledge of, and attitude towards influenza vaccination in Freetown Sierra Leone.

**Method:**

A cross-sectional study was conducted between February and April 2016 among healthcare providers working in four public and two private health facilities in Freetown Sierra Leone. Linear regression analysis, one-way ANOVA and independent t-test were employed for data analysis.

**Results:**

Among 706 respondents that participated in the study more than half were females 378 (53.6%), nurses 425 (60.4%), and the majority were between the age group of 20-39 years 600 (85.3%). Only 46 (6.5%) were vaccinated against influenza. Key reasons for not vaccinated against influenza were less awareness about influenza vaccination among HCPs 580 (82.73%) with (β = 0.154; CI 0.058–0.163), the high cost of influenza vaccines and therefore not normally purchased 392 (55.92%) having (β = 0.150; CI 0.063–0.186). More than half believed that HCPs are less susceptible to influenza infections than other people. Also, majority 585 (84.3%) of HCPs thought that influenza disease could be transmitted after symptoms appear. In addition, 579 (83.2%) of HCPs felt that symptoms usually appear 8–10 days after exposure. Close to half 321 (46.0%) of HCPs were not aware of the influenza immunisation guidelines published by the Advisory Committee on Immunization Practices and Centre for Disease Control.

**Conclusion:**

Influenza vaccine coverage among healthcare professionals in Freetown Sierra Leone was low. High cost, inadequate knowledge about influenza and its vaccine as well as the lack of awareness of vaccine availability were key barriers. Increasing access to influenza vaccine and the use of appropriate educational interventions to increase knowledge and awareness are required to improve influenza vaccination coverage among HCPs.

**Electronic supplementary material:**

The online version of this article (10.1186/s12889-017-4700-2) contains supplementary material, which is available to authorized users.

## Background

As a cause of high morbidity and mortality especially among vulnerable groups, influenza is recognised as a public health threat [1]. Vaccination against this disease is considered an effective infection control strategy [2]. For instance, influenza vaccines are known to be 60% protective among healthy people and decrease illness duration and severity in symptomatic individuals [3]. Because of their working environment, healthcare professionals (HCPs) are known to be at risk of contracting influenza virus from their patients and at the same time, act as a source of transmission to patients under their care. Such nosocomial transmission has the potential to trigger an epidemic within the hospital setting [4, 5]. The risk of transmission increases when HCPs continue to work after fallen sick [5]. Therefore, influenza vaccination does not only protect HCPs and thus contribute to maximizing their productivity [6], it also prevents patients from becoming sicker by providing additional protection by herd immunity [7–9]. In recognition of this fact, health institutions such as the World Health Organisation and the Advisory Committee on Immunization Practices (ACIP) recommend that HCPs should be vaccinated [10, 11]. Rigorous immunisation campaigns targeting vulnerable groups like HCPs have been conducted to prevent influenza-related illness and deaths in the healthcare setting [4, 12]. Despite such public health interventions, influenza vaccination coverage among HCPs around the world continues to be low [13–19]. Reasons for the low uptake of influenza vaccine among HCPs are the unavailability of the vaccine, misconception of vaccine safety and effectiveness and inadequate knowledge about the disease [16, 17, 20–23]. A clear insight of the limiting factors responsible for low vaccination coverage will contribute to developing workable interventions that will help increase influenza vaccine acceptance and uptake among HCPs.

In Sierra Leone, acute respiratory infections are one of the leading causes of morbidity and mortality [24]. The paucity of epidemiological data, especially on influenza has been a huge challenge to fully understand its public health impact. Notwithstanding, in 2011, the World Health Organization funded the Strengthening Influenza Sentinel Surveillance in Africa (SISA) project established a national influenza sentinel surveillance system integrated within the existing national integrated disease surveillance and response (IDSR) system [25]. Between the months of August and December 2011, 12.9% and 6.7% of all consultations in the sentinel sites accounted for influenza-like illness and severe respiratory infections respectively. Of the 473 samples collected, 12.7% tested positive for influenza virus with 55 (91.7%) were of the (H3N2) subtype and 6.7% had H1N1 subtype A [25]. Currently, influenza vaccination is not part of the national routine immunization program, and hitherto, there is no guideline on influenza vaccination for HCPs in Sierra Leone [26]. Also, there is the absence of research evidence that specifically looks at HCPs’ attitude and knowledge towards influenza vaccination. Therefore, this study was conducted to assess HCPs’ current influenza vaccine uptake rate, reasons for not getting vaccinated as well as their awareness, knowledge of, and attitude about influenza vaccination in Freetown Sierra Leone.

## Method

### Study design

A cross-sectional study was carried out among the HCPs practising at public and private hospitals in Freetown between February and April 2016.

### Study location

The study was conducted in Freetown, the capital city of Sierra Leone located along the West coast of Africa. We collected data for this study from HCPs in four public hospitals (Connaught teaching hospital, Princess Christian maternity hospital, Ola During paediatric hospital and 34 military hospitals) and two private hospitals (Chiothrams Memorial Hospital and Davidson Nicol Medical Centre). These health facilities were chosen to get a representative sample of all HCPs working in Freetown.

### Ethical approval

Ethical approval was obtained from the research and ethics committee of the College of medicine and health sciences, University of Sierra Leone.

### Sampling method

A convenience sampling method was adopted in which all HCPs working at these hospitals were invited to participate in the study. Five trained researchers were responsible for administering the questionnaires. The completed questionnaires were collected from the participants on the same day. Participation in the study was voluntary. Verbal consent was obtained from all participants after the rationale of the study was explained. We solicited verbal consent because the study did not pose any risk to participants. The research and ethics committee of the College of Medicine and Allied Health sciences, University of Sierra Leone approved such method of seeking consent to participate in the study.

### Questionnaire items

A previously validated survey tool (Cronbach’s alpha = 0.87) that addressed the same research question [16] was used (Additional file 1). The questionnaire comprised of 31-items. It comprised of questions that looked at respondent’s demographics and their vaccination status with regards influenza or any other diseases. It also questions that inquire about the reasons for not vaccinating against influenza using a five - point Likert scale. In addition, the questionnaire assessed the knowledge about influenza and influenza vaccines using a nominal scale (correct and incorrect) to measure responses.

### Statistical analysis

We coded all data from all completed questionnaires into nominal and ordinal variables. We used SPSS® version 22 for data analysis. Frequency and percentages were used to represent categorical variables. Linear logistic regression was employed to identify significant factors limiting influenza vaccination uptake (Q9-Q16) and those affecting knowledge and awareness (Q17-Q19) using profession as the covariate. Gender, years of experience and age were not included in this analysis due to wide confidence interval ranges. For question items (Q21-Q31), correct and incorrect responses for each statement was scored 1 and 0 respectively. Using summation of knowledge score as the dependent variable, and gender, profession and job experience as independent variables, one-way ANOVA test and independent sample t-test were used to determine contributing factors affecting the knowledge. A *p*-value less than 0.05 was considered statistically significant.

## Results

Among the health professionals (*N* = 706) approached for this survey, most were nurses *N* = 425 (60.4%), followed by Pharmacy Technicians 103 (14.6%) and physicians 46 (6.5%). More than half were females *N* = 378 (53.6%), between the age group 20-39 years 600 (85.3%). Close to half of HCPs, 349 (49.6%) had worked for 3–5 years. Only 108 (15.4%) of HCP’s were vaccinated in past 6–12 months for other diseases. Only 46 (6.5%) were vaccinated for influenza. Details are shown in Table 1.

**Table 1:** Demographics of respondents (*N* = 706)

Demographics	N (%)
Gender	
Male	327 (46.4)
Female	378 (53.6)
Age	
Range	20 - >50 years
20–29 years	304 (43.2)
30–39 years	296 (42.1)
40–49 years	77 (11.0)
> 50 years	26 (3.7)
Profession	
Physicians	46 (6.5)
Pharmacist	23 (3.3)
Pharmacy Technicians	103 (14.6)
Nurse	425 (60.4)
Nutritionist	32 (4.5)
Laboratory Technicians	27 (3.8)
Physiotherapist	9 (1.3)
Laboratory specialist	38 (5.4)
Community health officer	1 (0.1)
Education	
Certificate in Nursing	288 (40.8)
Diploma in Nursing	134 (19.0)
Diploma in Pharmacy	105 (14.9)
Diploma in medical laboratory science	63 (8.9)
MBBS/MD	44 (6.4)
Diploma in Nutrition	22 (2.8)
Bachelor of Pharmacy	23 (3.3)
Bachelor of Nursing	2 (0.2)
Certificate Nutrition	7 (0.9)
Bachelor of Nutrition	3 (0.4)
Diploma Physiotherapy	5 (0.7)
Certificate Physiotherapy	4 (0.5)
Job Experience	
1–2 years	239 (34.0)
3–5 years	349 (49.6)
6–10 years	77 (11.0)
> 10 years	38 (5.4)
Employment sector	
Private	100 (14.2)
Government	602 (85.5)
Others	2 (0.3)
Vaccination done in last 6–12 months for other diseases	
Yes	108 (15.4)
No	595 (84.6)
Name the disease for which you were vaccinated for?	
Yellow fever	54 (7.06)
Hepatitis B virus	28 (3.96)
Tetanus toxoid	24 (3.39)
Cholera	1 (0.14)
Elephantiasis	1 (0.14)
Vaccination done for Influenza	
Yes	46 (6.5)

Table 2 presents HCPs’ reasons for not being vaccinated against influenza using linear logistic regression. Linear logistic regression was applied with HCPs’ responses to the statements in Tables 2 and 5 taken as the dependent variables and profession taken as the independent variable. Beta coefficient (β) was used to compare the strength of the effect of dependent variables (reasons for not vaccinating against influenza) among the various cadres of HCPs (independent variable). The higher the β value, the stronger the relation. Physicians were set as a reference to compare the reasons for not vaccinating against influenza among health care professionals. Compared to physicians, other HCPs were 15% more likely not to get immunized against influenza due to the high cost of flu vaccine (β = 0.150; CI 0.063–0.186; *p* < 0.05) and their unfamiliarity with influenza vaccination (β = 0.154; CI 0.058–0.163; p < 0.05). Although statistically insignificant, insufficient staff to administer vaccine lack of proper storage area for influenza vaccines and concerns about vaccine safety were others barriers to influenza vaccine uptake among health care professional’s (details are shown in Table 2).

**Table 2:** Reasons for not vaccinating against influenza using profession as the independent variable

Statements	SA	A	DNK	D	SD	β	CI
There is lack of proper storage area for vaccines that’s why influenza vaccines is not available in the institution	187(26.7)	138(19.7)	142(20.3)	168(24.0)	65(9.3)	0.047	−0.25-0.113
It is not compulsory for health care professionals to get vaccinated for influenza	103(14.7)	194(27.7)	91(13.0)	209(29.8)	104(14.8)	0.071	−0.003-0.134
Influenza is not serious condition therefore not worth vaccinating	50(7.2)	83(11.9)	87(12.5)	352(50.5)	124(17.8)	−0.061	−0.173-0.017
Influenza vaccines is costly that’s why not purchased normally	145(20.7)	247(35.3)	150(21.4)	104(14.9)	54(7.7)	0.150*	0.063–0.186
Not everyone is familiar with influenza vaccination	227(32.8)	353(50.0)	49(6.9)	36(6.9)	35(5.0)	0.154*	0.058–0.163
There is insufficient staff to administer vaccine	114(16.3)	118(16.9)	132(18.7)	270(38.6)	64(9.2)	0.037	−0.033-0.097
Side effects and safety concerns are hindering health care professionals to get vaccinated for influenza	115(16.5)	223(31.9)	158(22.6)	158(22.6)	45(6.4)	−0.037	−0.091-0.031
I don’t like needles	174(24.9)	285(40.8)	33(4.7)	143(20.5)	63(9.0)	−0.015	−0.082-0.054

Logistic regression, * *p*-value <0.05 significant (Ref; physicians). (Other professions are shown in Table 1)

Addressing the knowledge of HCPs regarding influenza and its vaccine, 364 (51.6%) responded that HCPs are less susceptible to influenza infections than other people. Also, 585 (84.3%) HCPs believed that influenza could be transmitted after their symptoms appear. Also, 526 (75.6%) thought that the flu shot contains live viruses that may cause some people to get influenza. The majority of 454 (65.3%) believe that influenza is transmitted primarily by contact with blood and body fluids. Further, 579 (83.2%) of HCPs thought influenza disease has 8–10 days incubation period. See Table 3 for details.

**Table 3:** Knowledge about influenza and the influenza vaccine

Statements	Correct	Incorrect
Q21. Health care professionals are less susceptible to influenza infections than other people	364 (51.6)	332 (47.6)^a^
Q22. Influenza is transmitted primarily by coughing and sneezing	614 (88.3)^a^	82 (11.7)
Q23. Influenza is more serious than a “common cold	608 (87.2)^a^	89 (12.6)
Q24. The signs and symptoms of influenza include fever, headache, sore throat, cough, nasal congestion, and aches and pains	620 (89.1)^a^	76 (10.9)
Q25. HCPs can spread influenza even when they are feeling well	311 (44.7)^a^	385 (55.3)
Q26. People with influenza can transmit the infection only after their symptoms appear	585 (84.3)	111 (15.7)^a^
Q27. Influenza is transmitted primarily by contact with blood and body fluids	454 (65.3)	241 (34.7)^a^
Q28. The flu shot contains live viruses that may cause some people to get influenza	526 (75.6)	170 (24.4)^a^
Q29. Influenza vaccination does not work in some persons, even if the vaccine has the right mix of viruses	427 (61.4)^a^	268 (38.5)
Q30. Adults with influenza commonly experience nausea and vomiting or diarrhoea	311 (44.7)	385 (55.3)^a^
Q31. Symptoms typically appear 8–10 days after a person is exposed to influenza	579 (83.2)	117 (16.8)^a^

^a^Correct statement

To investigate the HCP’s knowledge about Influenza vaccination, correct responses were scored. Physicians had a better knowledge and understanding of influenza vaccine (5.8 ± 1.51), followed by nurses (5.7 ± 1.34), pharmacist (5.7 ± 1.65). However, HCPs with 6–10 years of job experience have better knowledge (6.1 ± 1.25) as compared to others. Also, females had a better understanding (5.7 ± 1.14) as compared to male HCPs. A detailed comparison of profession, experience and gender with knowledge score is shown in Table 4.

**Table 4:** HCP score for Knowledge about Influenza vaccination

Variable	Mean ± SD	p-value
Profession		
Physicians	5.8 ± 1.51	0.64 ^a^
Pharmacist	5.7 ± 1.65	
Pharmacy technicians	5.2 ± 1.31	
Nurse	5.7 ± 1.34	
Nutritionist	5.2 ± 1.21	
Laboratory Technicians	5.7 ± 1.25	
Physiotherapist	5.6 ± 1.35	
Laboratory specialist	5.7 ± 1.35	
*Community health officer	–	
Experience		
1–2 years	5.4 ± 1.30	0.001 ^a^
3–5 years	5.6 ± 1.40	
6–10 years	6.1 ± 1.25	
> 10 years	5.9 ± 1.27	
Gender		
Male	5.5 ± 1.30	0.089 ^b^
*Female*	5.7 ± 1.14	

*Only one community officer participated in the study, therefore, it was not possible to calculate the mean score and SD

a One-way ANOVA test

b Independent sample t-test

**Fig. 1:**
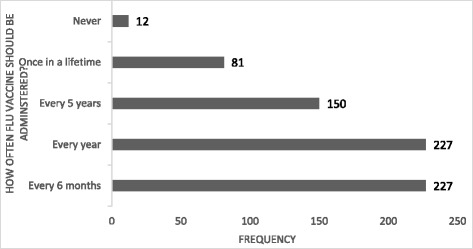
Responses on how often Flu vaccine should be administered?

To further investigate differences in knowledge about influenza vaccine among HCPs we used linear logistic regression model (Q17-Q19) against the profession. Overall, other professions have less knowledge about influenza vaccination as compared to the physicians. Compared to physicians, other cadres were less aware of the guidelines published by the Advisory Committee on Immunisation Practices (ACIP) or Centre for Disease Control (CDC) for influenza immunisation β = 0.154 [0.038–0.107]. Details are presented in Table 5. On how often influenza vaccine should be administered, an equal number of HCPs thought that influenza vaccine is to be administered every year and for every six months respectively (see Fig. 1).

**Table 5:** Knowledge about influenza vaccination against profession

Statements	Yes	No	Not sure	β	CI
Do you think the influenza vaccine is effective in preventing the ‘flu?	574(82.2)	63(9.0)	61(8.7)	0.074	0.000–0.063
Do you believe that the Centre for Disease Control (CDC) recommends that health care workers receive the flu shot?	378(54.2)	160(22.9)	160(22.9)	0.052	−0.013-0.072
Are you aware of the guidelines published by the Advisory Committee on Immunization Practices (ACIP) or Centre for Disease Control (CDC) for influenza immunization?	288(41.3)	321(46.0)	89(12.8)	0.154*	0.038–0.107

Logistic regression, * *p* -value <0.05 significant (Ref; physicians)

## Discussion

To our knowledge, this is the first ever type of study in Sierra Leone and perhaps in Africa that looks at influenza vaccination status among healthcare workers as well as their knowledge and perception regarding influenza disease and vaccination**.** As influenza is recognised as a public health challenge, health care providers are considered as vulnerable groups and vectors of transmission. Influenza immunization in the healthcare setting has been shown to be beneficial in reducing patient and health provider morbidity and mortality and as well as productivity [7, 27, 28].

Analysis of our data revealed a low influenza vaccine uptake by HCP (6.5%). Our result is generally in line with other studies done elsewhere although much lower [18–21, 23, 29–31] but slightly higher than the result reported by Khan et al. [16] and Bali et al. [22]. The high cost of the vaccine, lack of awareness among HCP about of influenza vaccine and concerns about influenza vaccine safety were the key reasons for low flu vaccine rate put forward by respondents in this study. Similar reasons were reported elsewhere [20–22]. The reasons mentioned above are in contrast to the results reported in studies conducted in Pakistan [16], Qatar [15] and Saudi Arabia [17] in which fear of needle and fear of contracting the illness and misconception of not being at risk were the key reasons put forward by participants respectively. Fear of side effects of the vaccine, its ineffectiveness to prevent the disease, and lack of time are other reasons that have been reported in other studies [17, 20, 31, 32]. Although the lack of knowledge and scepticism about influenza vaccine safety and efficacy are reasons for low vaccine uptake shared by participants in our study and those conducted elsewhere, our own was also based on inaccessibility due to the high cost of the vaccine. A study in Kenya reported a discordance between willingness to be vaccinated and acceptance levels of HCPs towards the vaccine [33]. Further studies should investigate whether HCP’s willingness to be immunised is translated into higher vaccine uptake. However, considering HCP are in short supply in Sierra Leone [34], the need to safeguard and maximize their health and productivity is of importance in ensuring the health of its citizenry is improved. Programs that increase influenza vaccine accessibility and affordability, as well as educational interventions to improve HCPs’ awareness about influenza vaccination in Sierra Leonean hospitals, need to be considered.

As it has been reported in the literature [17, 20, 22, 35], our study has demonstrated knowledge gap among HCPs about influenza and its vaccine. Although high cost and low level of awareness were cited as key reasons for low vaccine uptake, awareness of the disease and the misconception of the vaccine might be a contributing factor. In our study, more than half of HCPs believed that they were less susceptible to influenza virus than non-HCP. Also, most of them were also of the notion that influenza can be only be transmitted by symptomatic patients. These results are similar to a study conducted in Pakistan [16] but in shape contrast to what was reported in Saudi Arabia [21]. Respondents in our study also believed that influenza vaccine could cause influenza- a notion shared by other HCPs in other countries [16, 21, 22, 35]. Also, most (84.3%) of HCP in our study believed that only symptomatic individuals could transmit the disease mirroring finding from a Turkish study [32] but contrasted with results from Saudi Arabia and Pakistan [16, 21]. According to the CDC, the symptoms of influenza usually appear 1—4 days (average: 2 days) [36]. However, most participants (83.2%) in our study lack knowledge about the incubation period of the virus which is inconsistent with findings from Pakistan [16] and Saudi Arabia [21]. The deficit in knowledge in our study was also observed as the majority of HCPs did not know how often influenza vaccine should be administered which is inconsistent with a result of a similar study authored by Alshammari et al. [21]. With such knowledge deficit and low awareness, education interventions such training of HCPs through continuous professional development with help reduce the gap in knowledge and increase influenza vaccine acceptance and uptake.

Inferential statistics indicate that only years of experience was significantly associated with knowledge about influenza and vaccine when knowledge items were summed. HCPs with 6–10 years of experience were more knowledgeable than those with 1–2 years experience based on the scored eleven knowledge questions items. This result contrasts with the study conducted by Kahn et al. in which HCP with 1-2 years experience were more knowledgeable compared to those with more than two years experience. The same study also reported that nurses and physiotherapist were more knowledgeable than the other cadres of health professionals [16]. The fact that HCPs with less working experience in our study exhibited a deficiency in knowledge might be due to the possibility that modules on influenza were not properly taught in college or this cohort of HCPs have had less encounter with patients with or exposure to influenza and influenza-related symptoms. Also, it might be linked to the perception that the disease is self-limiting, and being young offers immunity. Less awareness of the influenza vaccine might affect HCPs’ uptake even if the vaccine is made available. Previous research has reported a positive link between increased knowledge of influenza and its vaccine and higher vaccine uptake among HCPs [17]. It is imperative that the need for increased education about influenza and its vaccine through training be intensified during pre- graduation and immediately after post-graduation. Our study also reported no significant difference in knowledge among the different groups of health care workers – a result that is not consistent with a similar study conducted in Pakistan in which nurses were more knowledgeable than physicians [16]. The difference observed may be linked to variations in study methodology and sample size. Ours was a multi-site study with a larger sample size as compared to that in Pakistan which was quite the opposite. With regards to gender, our study resonates with what was observed in Pakistan [16]. Also, HCPs further demonstrated a lack of knowledge and awareness of influenza vaccination in that more than half of the HCPs surveyed were not aware or were not too sure of CDC’s recommendation for mandatory influenza vaccination among all healthcare providers. We observed similar response with regards to their awareness of the joint CDC and ACIP guidelines on influenza vaccination. Previous research has reported similar observation [21]. It is essential that appropriate education strategies are developed to increase knowledge and awareness among HCPs in Freetown Sierra Leone.

### Limitations and strength

A key limitation of this study is that our findings cannot be generalised for the whole country as we surveyed only healthcare professionals in Freetown. Future studies should incorporate a nationwide sample to get a representative view of HCP country wide regarding influenza vaccination. Also, the sampling method used reduces the possibility every HCP to have an equal chance of being selected. However, this sampling method was chosen to target key respondents that can provide meaningful information required to sufficiently test the study hypothesis. Notwithstanding these limitations, a key strength of our study was that we used a large sample size and HCPs were targeted both from both public and private health facilities.

## Conclusion

Influenza vaccine uptake among HCPs in Freetown Sierra Leone is very low. High cost, inadequate knowledge about influenza and its vaccine as well as the lack of awareness of vaccine availability were key reasons for the low coverage. Increasing access to influenza vaccine and the use appropriate educational interventions to increase knowledge and awareness are required to improve influenza vaccination coverage among HCP.

## Additional files

Additional file 1: A cross sectional survey on health care professional’s awareness of knowledge about and attitude towards influenza vaccination in Freetown Sierra Leone. (DOCX 115 kb)

Additional file 2: SPSS dataset. (SAV 851 kb)

## Abbreviations

HCP: Healthcare professionals
IDSR: Integrated Disease Surveillance and Response
SISA: Sentinel Surveillance in Africa
