# Supplementary material for: An assessment of healthcare professionals’ knowledge about and attitude towards influenza vaccination in Freetown Sierra Leone: a cross-sectional study
Source: BMC Public Health. 2017 Sep 5;17:692. doi: 10.1186/s12889-017-4700-2 (PMC5584505; doi:10.1186/s12889-017-4700-2)
Supplement: Supplementary file 1 — A cross sectional survey on health care professional’s awareness of knowledge about and attitude towards influenza vaccination in Freetown Sierra Leone. (DOCX 115 kb) [file 12889_2017_4700_MOESM1_ESM.docx]

A CROSS SECTIONAL SURVEY ON HEALTH CARE PROFESSIONAL’S AWARENESS OF KNOWLEDGE ABOUT AND ATTITUDE TOWARDS INFLUENZA VACCINATION IN FREETOWN SIERRA LEONE

Please give a moment to complete the survey by answering all the questions.
Purely for research purpose.
No conflict of interest.

Top of Form

**1) Gender**

- - Male
  - Female

**2) Profession**

- - o Physician
  - o Pharmacist
  - o Nurse
  - o Nutritionist
  - o Lab. Specialist
  - o Physiotherapist

**3) Qualification**

**4) Age**

**5) Job experience in years**

- - 1 to 2 years
  - 3- 5 years
  - 6- 10 years
  - More than 10 years

**6) Employment sector**

- - Private
  - Government
  - Semi government
  - Others ( please specify)
  - Other: 

**7) In last 6-12 months, have you vaccinated yourself against any disease**

- - Yes
  - No
  - Never vaccinated in last 2-3 years’ time

**If yes to Q 7 please provide the name of vaccine**

**8) In last 6-12 months have you vaccinated yourself against influenza**

- - Yes
  - No
  - o Never vaccinated in last 2-3 years’ time

**9) There is lack of proper storage area for vaccines that’s why Influenza vaccines is not available in the institution**

Reasons for not vaccinating against influenza Kindly share your opinion.

- - Strongly Agree
  - Agree
  - Don't know
  - Disagree
  - Strongly Disagree

**10) It is not compulsory for health care professionals to get vaccinated for Influenza**

Reasons for not vaccinating against influenza Kindly share your opinion.

- - Strongly Agree
  - Agree
  - Don't know
  - Disagree
  - Strongly Disagree

**11) Influenza is not serious condition therefore not worth vaccinating**

Reasons for not vaccinating against influenza Kindly share your opinion.

- - Strongly Agree
  - Agree
  - Don't know
  - Disagree
  - Strongly Disagree

**12) Influenza vaccines is costly that’s why not purchased normally**

Reasons for not vaccinating against influenza Kindly share your opinion.

- - Strongly Agree
  - Agree
  - Don't know
  - Disagree
  - Strongly Disagree

**13) Not everyone is familiar with Influenza vaccination**

Reasons for not vaccinating against influenza Kindly share your opinion.

- - Strongly Agree
  - Agree
  - Don't know
  - Disagree
  - Strongly Disagree

**14) There is insufficient staff to administer vaccine**

Reasons for not vaccinating against influenza Kindly share your opinion.

- - Strongly Agree
  - Agree
  - Don't know
  - Disagree
  - Strongly Disagree

**15) Side effects and safety concerns are hindering health care professionals to get vaccinated for influenza**

Reasons for not vaccinating against influenza Kindly share your opinion.

- - Strongly Agree
  - Agree
  - Don't know
  - Disagree
  - Strongly Disagree

**16) I don’t like needles**

Reasons for not vaccinating against influenza Kindly share your opinion.

- - Strongly Agree
  - Agree
  - Don't know
  - Disagree
  - Strongly Disagree

**17) Do you think the influenza vaccine is effective in preventing the ‘flu?**

Health care professionals (HCPs) general understanding about the influenza vaccine. Kindly share your opinion.

- - Yes
  - No
  - Not sure

**18) Do you believe that the Centre for Disease Control (CDC) recommends that health care workers receive the flu shot?**

Health care professionals (HCPs) general understanding about the influenza vaccine. Kindly share your opinion.

- - Yes
  - No
  - Not sure

**19) Are you aware of the guidelines published by the Advisory Committee on Immunization Practices (ACIP) or Centre for Disease Control (CDC) for influenza immunization?**

Health care professionals (HCPs) general understanding about the influenza vaccine. Kindly share your opinion.

- - Yes
  - No
  - Not sure

**20) How often do you think the flu vaccine should be administered?**

Health care professionals (HCPs) general understanding about the influenza vaccine

- - Every 6 months
  - Every year
  - Every 5 years
  - Once in a lifetime
  - Never

**21) Health care professionals are less susceptible to influenza infections than other people**

Awareness Of HCPS About Influenza And The Influenza Vaccine. Kindly share your opinion

- - Correct
  - Incorrect

**22) Influenza is transmitted primarily by coughing and sneezing**

Awareness Of HCPS About Influenza And The Influenza Vaccine. Kindly share your opinion

- - Correct
  - Incorrect

**23) Influenza is more serious than a “common cold**

Awareness Of HCPS About Influenza And The Influenza Vaccine. Kindly share your opinion

- - Correct
  - Incorrect

**24) The signs and symptoms of influenza include fever, headache, sore throat, cough, nasal congestion, and aches and pains**

Awareness Of HCPS About Influenza And The Influenza Vaccine. Kindly share your opinion

- - Correct
  - Incorrect

**25) HCPs can spread influenza even when they are feeling well.**

Awareness Of HCPS About Influenza And The Influenza Vaccine. Kindly share your opinion

- - Correct
  - Incorrect

**26) People with influenza can transmit the infection only after their symptoms appear**

Awareness Of HCPS About Influenza And The Influenza Vaccine. Kindly share your opinion

- - Correct
  - Incorrect

**27) Influenza is transmitted primarily by contact with blood and body fluids**

Awareness Of HCPS About Influenza And The Influenza Vaccine. Kindly share your opinion

- - Correct
  - Incorrect

**28) The flu shot contains live viruses that may cause some people to get influenza**

Awareness Of HCPS About Influenza And The Influenza Vaccine. Kindly share your opinion

- - Correct
  - Incorrect

**29) Influenza vaccination does not work in some persons, even if the vaccine has the right mix of viruses**

Awareness Of HCPS About Influenza And The Influenza Vaccine. Kindly share your opinion

- - Correct
  - Incorrect

**30) Adults with influenza commonly experience nausea and vomiting or diarrhea**

Awareness Of HCPS About Influenza And The Influenza Vaccine. Kindly share your opinion

- - Correct
  - Incorrect

**31) Symptoms typically appear 8–10 days after a person is exposed to influenza**

Awareness Of HCPS About Influenza And The Influenza Vaccine. Kindly share your opinion

- - Correct
  - Incorrect
